# Development and evaluation of curcumin nano-niosomes for glioma-targeted therapy

**DOI:** 10.1038/s41598-025-95348-5

**Published:** 2025-03-27

**Authors:** Hao Qian, Jiaqi Lv, Xiuping Hu

**Affiliations:** 1 Department of Pharmacy, The Affiliated Chuzhou Hospital of Anhui Medical University, 369th Zhuwengxi Road, Nanqiao District, Chuzhou, 239000 China; 2 Tianjin University of Science and Technology, 1038th Dagu Nan Road, Hexi District, Tianjin, China

**Keywords:** Niosomes, Curcumin, Transferrin, Glioma, Brain targeting, Biochemistry, Cancer, Oncology, Materials science, Nanoscience and technology, Blood-brain barrier

## Abstract

Glioma remains a significant global health challenge, and is characterized by a persistently high mortality rate. Chemotherapy is a common treatment for glioma, but many anticancer drugs exhibit poor permeability across the blood–brain barrier (BBB) and fail to reach tumor tissues adequately, while also exerting toxic effects on normal cells. To address these issues, this study investigated the use of niosomes (Nio), which are biocompatible, biodegradable, and non-immunogenic, to encapsulate curcumin (Cur) and enhance its delivery to glioma tissues. Niosomes were prepared using the non-ionic surfactant sorbitan monostearate (Span 60) and cholesterol as carrier materials, and subsequently modified with transferrin (TF) to facilitate receptor-mediated transport across the BBB. The resulting TF-modified curcumin niosomes (TF-Cur-Nio) demonstrated enhanced targeting of brain tumors, improved anti-glioma efficacy, and favorable in vivo safety. These findings suggest that the TF-Cur-Nio delivery system has significant potential for advancing glioma treatment by overcoming the limitations of conventional chemotherapy and improving drug delivery to the brain.

## Introduction

Gliomas are among the most lethal malignant tumors, and are characterized by high incidence and mortality rates^1^. Despite the availability of primary treatment modalities such as surgical resection, radiotherapy, and chemotherapy^2–5^, these approaches are severely hindered by the blood-brain barrier (BBB) and their nonspecific nature, which leads to significant systemic toxicities. To surmount these challenges, there is an urgent need for innovative drug delivery systems that can precisely target glioma cells while minimizing damage to healthy tissues.

Currently, one of the primary obstacles to treating gliomas with chemotherapy is the BBB^6^. The BBB is a dynamic barrier between the blood and brain tissue and is considered one of the most complex and important organs in the human body. Its presence maintains a relatively stable internal environment in the brain, protecting the integrity and dynamic balance of the brain, preventing external damage and other harmful stimuli, and ensuring the normal physiological functions and nutrient transport of the central nervous system (CNS)^7^. Different substances enter the brain through the BBB via various mechanisms. The BBB allows various substances to cross it through different pathways: (a) diffusion through tight junctions, (b) lipid-soluble small molecule penetration transcellularly through the lipid plasma membrane, (c) carrier-mediated transport, (d) efflux pump-mediated removal of unwanted compounds, (e) specific receptor-mediated targeted transport, (f) adsorptive-mediated transcytosis, and (g) cell-mediated transcytosis^8^.

The presence of the BBB prevents almost 100% of large molecule drugs and more than 98% of small molecule drugs from entering the brain^8^. Thus, developing drug carriers capable of crossing the BBB is a significant challenge for treating brain diseases.

Nanoparticle-based drug delivery systems offer a promising, non-invasive approach to crossing the BBB for glioma treatment. Their customizable structures and surface properties enable targeted, controlled, and prolonged drug release in the brain. Additionally, these systems extend drug circulation in the bloodstream, reducing drug inactivation and enhancing bioavailability. Nanoparticles can also carry various drug types, including proteins and peptides, thus broadening their potential applications^9^.

Although the use of nanocarriers to traverse the BBB for glioma treatment is considered noninvasive and highly promising, practical implementation still requires careful adaptation to different administration routes. These include intravenous injection (IV), intranasal (IN) delivery, and localized brain injection^10^.

IV administration is a common systemic approach for glioma therapy. Under these conditions, nanocarriers generally need good circulation stability and a relatively small particle size (often 100–200 nm) to avoid rapid clearance by the mononuclear phagocyte system. Surface modifications such as PEGylation can also reduce protein adsorption, thereby enhancing the stealth effect^11^. IN administration takes advantage of the olfactory or trigeminal nerve pathways to deliver drugs directly to the brain, partially bypassing the BBB. However, carriers must have adequate mucoadhesive or mucopenetration properties and must remain biocompatible and soluble^12,13^. In some post-surgical or specialized settings, drugs can be administered directly to the tumor site via intracranial or convection-enhanced delivery. Although this approach avoids systemic distribution, it is more invasive, and uneven local distribution may occur. Consequently, higher demands are placed on carrier biodegradability and local retention^14^.

Beyond the route of administration, factors such as carrier size, surface charge, shape, hydrophilicity/lipophilicity, and active targeting ligands all influence distribution, uptake, and metabolism across the BBB^15–17^. For gliomas, which exhibit a unique tumor microenvironment, these considerations must also incorporate tumor vascular permeability and cerebrospinal fluid circulation. Selecting suitable nanomaterials and further functionalizing their surfaces with targeting ligands is thus crucial. Such designs allow for both passive targeting (via enhanced permeability and retention or BBB-related features) and active targeting (via receptor-mediated transport), ultimately enabling more efficient drug delivery to brain tumors.

Nanoparticle-mediated brain-targeted drug delivery relies primarily on two methods: passive targeting and active targeting^18,19^. Passive transport involves the natural permeation of nanoparticles across the BBB based on the physicochemical properties of the carrier, such as size, shape, and charge. P-glycoprotein (P-gp), located on the luminal side of brain capillary endothelial cells, acts as a molecular pump common on the BBB that protects cells from harmful foreign molecules. Conventional therapeutic drugs bound to P-gp tend to be pumped out of the cell membrane, whereas nonionic surfactants exhibit high affinity for P-gp and this interaction indirectly facilitates the transport of drugs across the BBB, thereby acting as inhibitors of P-gp on capillary endothelial cells^20,21^.

Most prior studies on brain-targeted nanocarriers rely on liposomes or PLGA nanoparticles. However, these systems often face challenges such as liposome aggregation during storage^22^ or complex PLGA synthesis requiring toxic solvents^23^. In contrast, nonionic surfactant vesicles (niosomes, Nio), composed of non-ionic surfactants and cholesterol, offer enhanced stability and a more straightforward, solvent-free preparation process, making them a promising alternative for brain-targeted drug delivery.

Nios are closed bilayer vesicles composed of a mixture of cholesterol and nonionic surfactants^24,25^. Like liposomes, Nios can encapsulate hydrophobic drugs within their hydrophobic membrane and hydrophilic drugs within their internal aqueous phase. Nios exhibit biodegradability, biocompatibility, non-immunogenicity, and low toxicity, along with ease of storage^26,27^. In recent years, Nios have been considered among the most promising nanocarriers for targeted drug delivery^26^.

The primary components of Nio include nonionic surfactants, hydration medium, and lipids. Nonionic surfactants are amphiphilic molecules that possess both hydrophilic and hydrophobic moieties^28^. Common nonionic surfactants used for Nio formation include alkyl ethers, alkyl esters, alkylamides, and fatty acids^29,30^. The nonionic surfactant component can promote the targeted delivery of therapeutic agents to the brain by inhibiting the efflux of P-gp, thereby enhancing their potential as a carrier for brain-targeted drug delivery.

On the other hand, active transport refers to specific molecular mechanisms or receptor-mediated approaches that enable the targeted delivery of nanoparticles and their associated therapeutic agents to the brain. To further enhance targeted delivery to glioma cells, the surface of drug carriers could be modified with targeting moieties that enable receptor-mediated transport. Currently, several surface receptors distinct from those found in other normal tissues, such as transferrin (TF), lipoprotein, or insulin-like growth factor receptors have been identified on the surfaces of brain capillary endothelial cells and glioma cells^31^. Modifying the surface of carriers with targeting ligands increases affinity for BBB or glioma cells, allowing specific binding to overexpressed targets on the cell surface. This binding triggers receptor-mediated endocytosis, reducing uptake by normal cells and thereby increasing the specificity of drug accumulation within the targeted tissues or intracellular organelles^32,33^. For example, Mishra et al. delivered the antiviral drug zidovudine via TF-modified PEGylated albumin nanoparticles. Their results revealed significantly greater brain accumulation of TF-modified nanoparticles than unmodified ones, demonstrating the feasibility of receptor-mediated nanocarrier transport to the brain^34^.

In this study, we developed an innovative brain-targeting drug delivery system that combines passive and active targeting strategies using TF-modified Nio to encapsulate curcumin (Cur). Cur, a polyphenolic compound derived from *Curcuma longa*, has demonstrated anticancer properties against various malignancies, including gliomas. However, its clinical application is limited due to poor water solubility, rapid degradation, and low bioavailability^35^.

Therefore, the use of different nanocarriers to encapsulate Cur can increase its efficacy as an anticancer agent, enabling it to penetrate the blood-brain barrier, prolong its release, and target delivery to tumor tissues. Nios are beneficial for promoting drug delivery to the brain and enhancing brain targeting, as the nonionic surfactants in the vesicle composition act as inhibitors of P-gp on capillary endothelial cells^36^. Thus, Nios can protect Cur from degradation and oxidation, improve its solubility and bioavailability, and enhance its therapeutic effectiveness in crossing the BBB and treating cancers such as gliomas. Additionally, their surfaces can easily be functionalized and modified with ligands to facilitate both passive and active targeting through the BBB^37^.

Our approach is distinct in that we functionalized the surface of Nio with TF ligands to achieve receptor-mediated targeting. Transferrin receptors (TFR) are overexpressed on both the BBB and glioma cells, facilitating enhanced drug delivery to the brain. The Nio-based delivery system introduces a dual-targeting strategy by combining passive BBB penetration (via P-gp inhibition by nonionic surfactants) and active glioma cell targeting (via TF conjugation). This dual-targeting strategy not only improves the therapeutic efficacy of curcumin against gliomas but also minimizes systemic side effects.

**Fig. 1:**
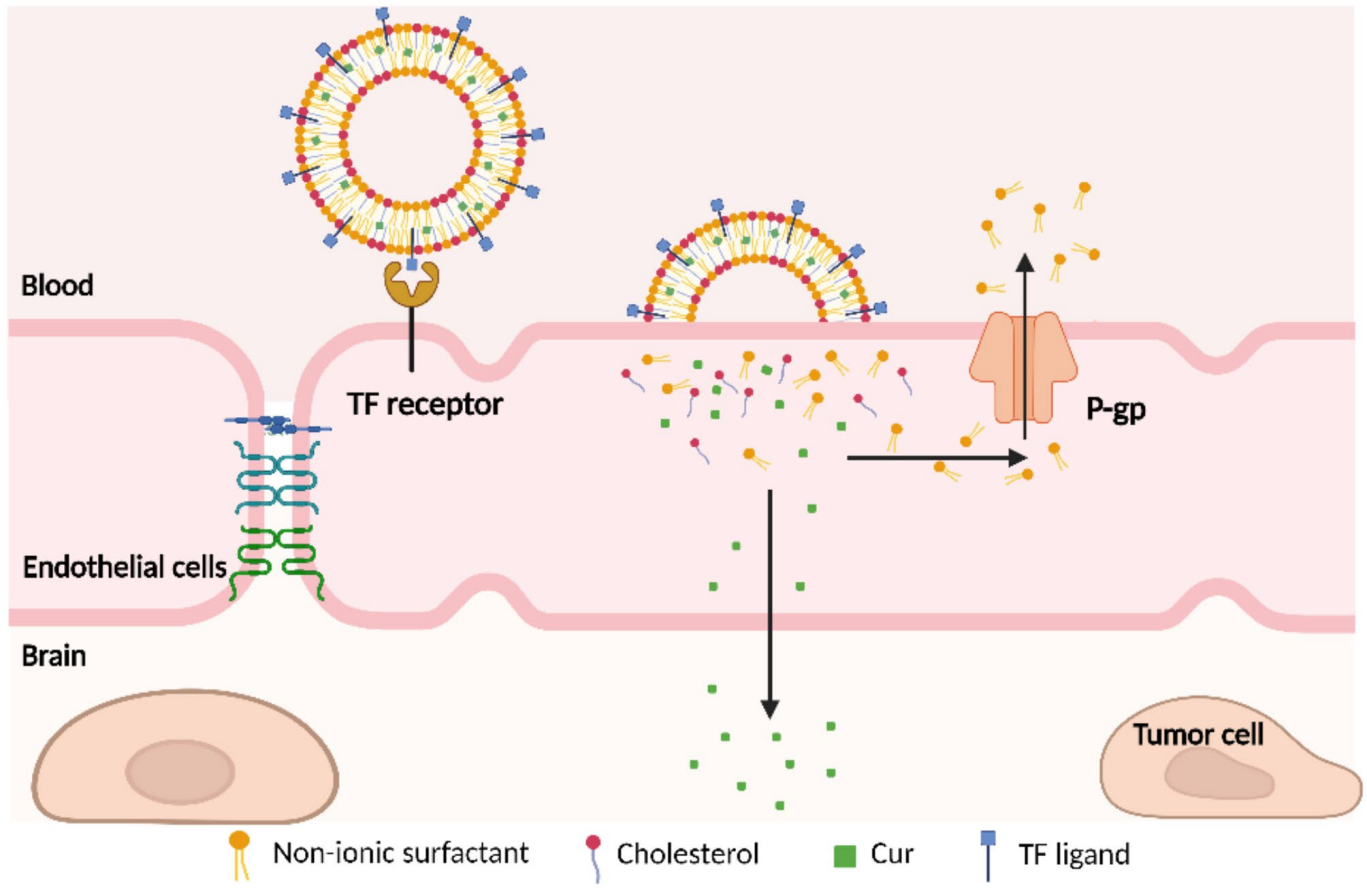
Diagram of the delivery of TF-Cur-Nio across the BBB. TF ligands on the Nio surface bind to TFR overexpressed on BBB endothelial cells and glioma cells, triggering receptor-mediated endocytosis and facilitating drug transport into the brain. The nonionic surfactant components of the Nios can help circumvent P-gp efflux, thereby enhancing curcumin accumulation within tumor tissues.

Here, as shown in Fig. 1, the surface of Nio was modified with a TF ligand for receptor-mediated targeting to the brain. TF receptors are highly expressed on the surface of the BBB and in glioma cells. Both the C-terminal and N-terminal regions of TF can interact with the helical domain and protease-like domain of TFR, leading to the binding of TF to TFR. This interaction facilitates the possibility of delivering drugs to the brain. This study designed and prepared TF-Cur-Nio for targeted therapy against glioma, effectively enhancing the brain tumor-targeting ability and anti-glioma efficacy of Cur. The system demonstrated good in vivo safety, indicating that the TF-Cur-Nio delivery system has potential for improving the treatment of glioma.

## Results

### Preparation and characterization of Cur-Nio/TF-Cur-Nio

**Fig. 2:**
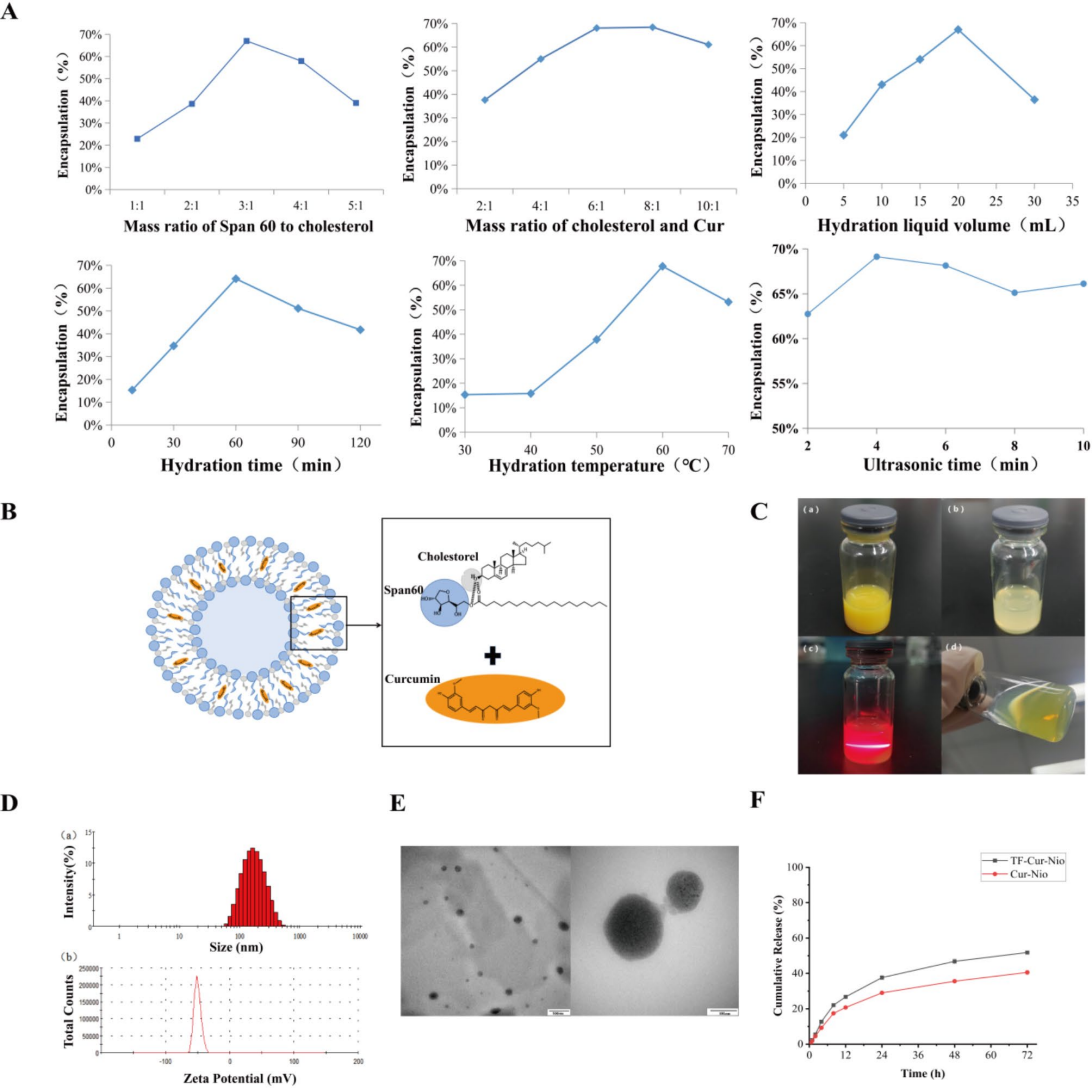
Preparation and characterization of Cur-Nio and TF-Cur-Nio. (**A**) Results of single-factor optimization experiments. (**B**) Schematic illustration of Cur-Nio formation, showing its key components (Span 60, cholesterol, and Cur). (**C**) Appearance of Cur-Nio suspensions: (a) original solution, (b) diluted solution, (c) Tyndall effect of diluted solution, and (d) fluorescence under UV light irradiation of diluted solution. (**D**) Particle size distribution (a) and zeta potentials (b) of TF-Cur-Nio. (**E**) TEM images of TF-Cur-Nio. (**F**) Cumulative release profiles of Cur-Nio and TF-Cur-Nio over time (n = 3).

Cur-Nio vesicles were prepared using a thin-film dispersion–ultrasonication method. Single-factor experiments were conducted to assess how the Span 60-to-cholesterol mass ratio, the cholesterol-to-curcumin mass ratio, the hydration volume, the hydration time, the hydration temperature, and the sonication time affected encapsulation efficiency (Fig. 2A).

For the Span 60 to cholesterol ratio, encapsulation efficiency initially increased and then declined. Too little Span 60 limited curcumin encapsulation, whereas excessive Span 60 combined with low cholesterol rendered the membrane unstable. The optimal ratio range was 2:1 to 4:1. Regarding the cholesterol-to-curcumin ratio, adding more cholesterol initially stabilized the vesicular membrane, but excessive amounts reduced its fluidity; a 6:1 ratio proved optimal.

Small hydration volumes led to poor dispersion and low efficiency, whereas overly large volumes also decreased efficiency; thus, 15–25 mL was deemed ideal. Insufficient hydration time produced incomplete vesicles, while prolonged hydration damaged them; 1.1 h was optimal. Hydration temperatures below 53 °C hindered vesicle formation, whereas 60 °C was optimal. Sonication time improved dispersion up to a point, but excessively long sonication disrupted vesicles; 4 min was identified as optimal.

Subsequently, a three-factor, five-level central composite design–response surface methodology (CCD–RSM) was applied to the key factors (Span 60-to-cholesterol ratio, hydration time, and hydration volume). Statistical analysis of the CCD–RSM results yielded final optimized conditions of a 3.10:1 Span 60-to-cholesterol ratio, a 1.1 h hydration time, and a 20.0 mL hydration volume. Under these conditions, Cur-Nio exhibited an average particle size of (160.83 ± 2.15)  nm, a PDI of 0.16 ± 0.04, a zeta potential of (− 44.13 ± 1.91) mV, and an encapsulation efficiency (EE) of (81.37 ± 0.62)%.

The formation and appearance of the optimized Cur-Nio are illustrated in Fig. 2B, C. The undiluted Cur-Nio appeared as a yellow suspension with no Tyndall effect, likely due to its high concentration. Upon dilution by a factor of ten, the Cur-Nio turned into a clear yellow solution that exhibited a faint blue opalescence under light and a distinct Tyndall effect when illuminated with infrared light. To assess its stability, Cur-Nio was stored at 4 °C for 30 days. After this period, the upper suspension remained visually unchanged, appearing uniform and stable. The results for particle size, PDI, zeta potential, EE, and drug loading (DL) are presented in Table 1. A slight decrease in encapsulation efficiency and drug loading was observed, while particle size, PDI, and zeta potential remained relatively stable. These findings suggest that the Cur-Nio suspension exhibited good stability over 30 days at 4 °C.

**Table 1:** Stability evaluation of Cur-Nio suspension at 4 °C over 30 days (n = 3).

Time (d)	Size (nm)	PDI	Zeta potential (mV)	Encapsulation efficiency (%)	Drug loading (%)
0	169.1	0.147	− 45.7	82.45 ± 0.43	2.59 ± 0.01
7	167.6	0.153	− 44.7	81.97 ± 0.50	2.57 ± 0.03
15	168.5	0.162	− 42.0	82.01 ± 0.61	2.53 ± 0.02
30	163.0	0.162	− 44.0	80.62 ± 0.47	2.51 ± 0.03

Cur-Nio was subsequently modified with TF to obtain TF-Cur-Nio. The measured average particle size of TF-Cur-Nio was (168.4 ± 0.75) nm, with a PDI of (0.154 ± 0.0070) and a zeta potential of (− 51.62 ± 6.12) mV (Fig. 2D). The strong negative surface charge indicates significant electrostatic repulsion, contributing to high stability^38^. Furthermore, UV absorption measurements using a TF standard curve (y = 0.0041x − 0.0151, R^2^ = 0.9993) confirmed that TF was conjugated onto the Nios with an efficiency of 87.63%, providing direct evidence that TF was successfully anchored on the vesicle surface. TEM images revealed that TF-Cur-Nio appeared as spherical vesicles with a structurally intact appearance, with curcumin encapsulated within the vesicle membrane. The vesicles exhibited smooth and uniform surfaces, and their observed size was consistent with the measured values (Fig. 2E).

Due to the poor solubility and instability of Cur under in neutral and alkaline conditions, 0.5% SDS phosphate buffer at pH 5 was used as the release medium. The release rate of TF-Cur-Nio was faster than that of Cur-Nio, with 51% released over 72 h. This increase in release rate was attributed to the insertion of TF, which may have loosened the vesicle structure. The in vitro drug release process of TF-Cur-Nio followed a first-order model (Fig. 2F). The release data fitted a first-order model (Table 2).

**Table 2:** Release model fitting results of TF-Cur-Nio.

Model	Fitting equation	r
Zero-order model	M_t_/M_∞_ = 0.698t + 9.6536	0.9104
First-order model	ln (1 − M_t_/M_∞_) = 0.0195t + 0.7906	0.9959
Higuchi model	M_t_/M_∞_ = 6.8981t^1/2 ^– 1.3634	0.9864
Weibull model	lnln[1/(1 − M_t_/M_∞_)] = 0.7005lnt + 0.5604	0.9700

In summary, the systematic optimization of Cur-Nio and subsequent modification with TF resulted in highly stable and efficient drug delivery systems. The optimized Cur-Nio demonstrated desirable physicochemical properties, including an appropriate particle size, PDI, zeta potential, and high EE. The introduction of TF not only increased stability but also accelerated the drug release rate, making TF-Cur-Nio a promising candidate for targeted drug delivery applications.

### Evaluation of Cur-Nio/TF-Cur-Nio at the cellular level

After the preparation and characterization of Cur-Nio and TF-Cur-Nio, we evaluated the performance of these two formulations at the cellular level.

**Fig. 3:**
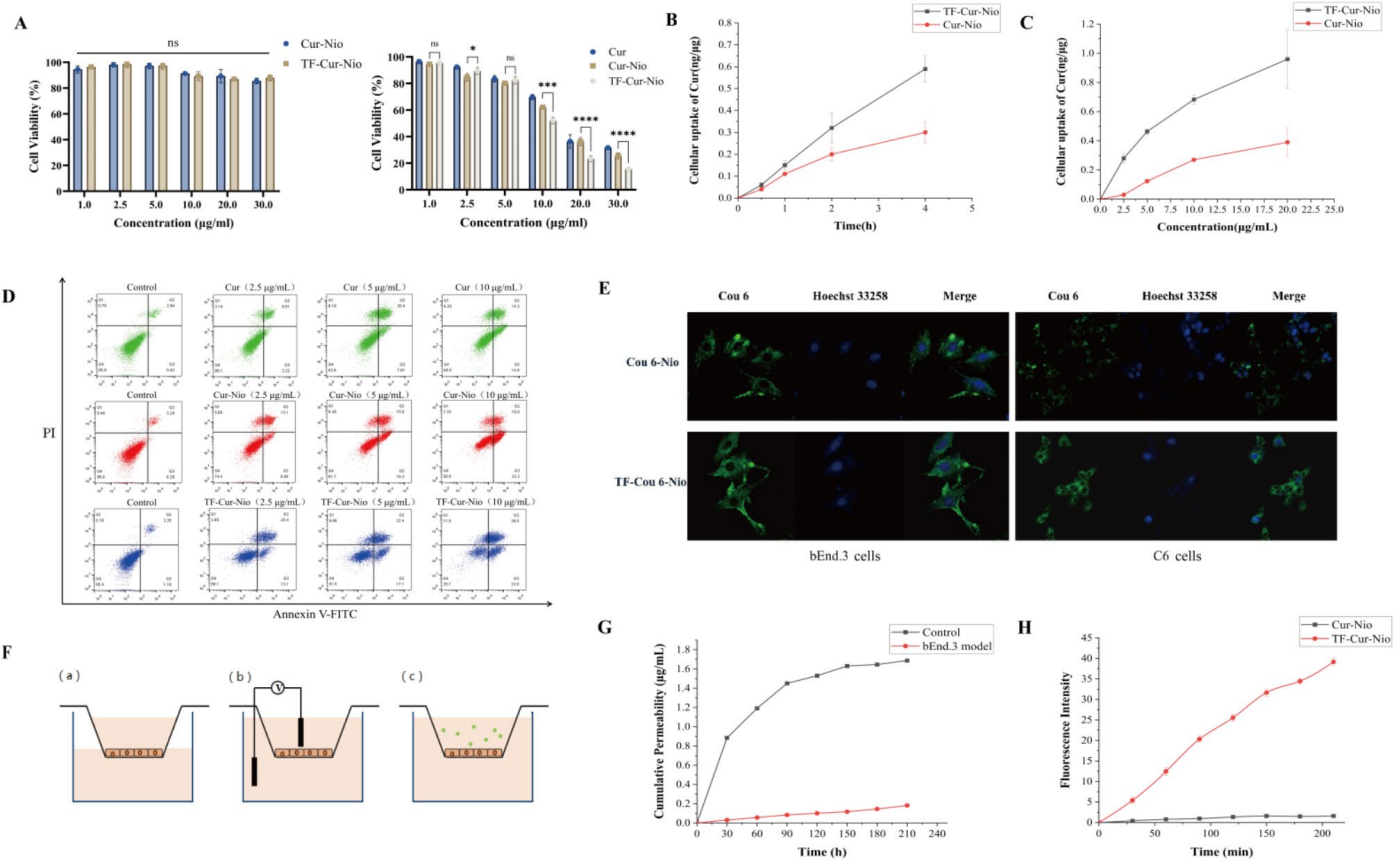
In vitro evaluation of Cur-Nio and TF-Cur-Nio. (**A**) Cell viability of (a) C6 glioma cells and (b) bEnd.3 endothelial cells after 24 h incubation with different concentrations of Cur, Cur-Nio, and TF-Cur-Nio (n = 5). Data was analyzed by unpaired t-test, and statistical significance was set at p* < 0.05. (**B**) Time-dependent cellular uptake of Cur in C6 cells after treatment with Cur-Nio and TF-Cur-Nio (n = 3). (**C**) Concentration-dependent cellular uptake of Cur in C6 cells after treatment with Cur-Nio and TF-Cur-Nio (n = 3). (**D**) Flow cytometry analysis of C6 cell apoptosis using Annexin V/PI double staining after treatment with different formulations (n = 3). (**E**) Fluorescence imaging of Cur-Nio and TF-Cur-Nio uptake in C6 and bEnd.3 cells. Green: Coumarin-6 (representing Cur); Blue: Hoechst 33,258 (nuclear staining). (**F**) Schematic diagram of the bEnd.3 cell model verification experiment: (a) 4 h leakage test across the membrane (b) TEER value measurement, and (c) sodium fluorescein permeability test. (**G**) Cumulative permeability of sodium fluorescein in the bEnd.3 cell model (n = 3). (**H**) Fluorescence intensity of Cou6 in the lower chamber of the bEnd.3 cell model (n = 3).

First, the cytotoxicity of Cur-Nio and TF-Cur-Nio was evaluated in bEnd.3 (normal) and C6 (cancer) cells via the MTT assay. In bEnd.3 cells, neither Cur-Nio nor TF-Cur-Nio showed significant toxicity with increasing Cur concentrations, indicating that Cur-Nio was safe for normal cells. In contrast, for C6 cells, cytotoxicity increased with the increase of Cur concentrations. The Cur-Nio group had similar cytotoxicity to that of free Cur, while TF-Cur-Nio exhibited the highest cytotoxicity because of enhanced cellular uptake via receptor-mediated endocytosis. The IC_50_ values (concentrations inhibiting 50% of cell growth) were: 15.89 μg/mL for Cur, 13.5 μg/mL for Cur-Nio, and 10.67 μg/mL for TF-Cur-Nio. These results indicated that TF-Cur-Nio significantly reduced the IC_50_ value, enhancing its cytotoxic effect on C6 cells. These findings provide a basis for further cell-based studies (Fig. 3A).

The cellular uptake of Cur-Nio and TF-Cur-Nio was subsequently evaluated at different time points and concentrations. Compared with those of Cur-Nio, the uptake of TF-Cur-Nio was significantly greater in C6 cells, with both formulations exhibiting time-dependent increases in uptake (Fig. 3B). At Cur concentrations ranging from 2.5 to 20 µg/mL, the uptake of TF-Cur-Nio by C6 cells was also significantly greater than that of Cur-Nio, and the uptake of both formulations increased in a concentration-dependent manner (Fig. 3C).

Next, using Annexin V-FITC/PI dual staining, we evaluated the effects of different concentrations of Cur, Cur-Nio, and TF-Cur-Nio on the apoptosis of C6 glioma cells. At 2.5, 5, and 10 μg/mL, the rates of apoptosis induced by the Cur solution were 13.87%, 34.45%, and 36.39%, respectively. For Cur-Nio, the rates were 25.69%, 38.29%, and 49.53%, whereas for TF-Cur-Nio, they were significantly higher at 41.95%, 48.49%, and 74.3%. Compared with Cur, TF-Cur-Nio markedly increased the percentage of apoptosis cells (P < 0.01) and significantly increased the percentage of proapoptotic cells (Fig. 3D). These findings highlighted the effectiveness of TF-Cur-Nio in inducing C6 glioma cell apoptosis.

Confocal laser scanning microscopy revealed that bEnd.3 cells treated with TF-Cur-Nio also presented stronger fluorescence intensity than those treated with Cur-Nio did, indicating enhanced uptake due to TF binding to the TFR on bEnd.3 cells. Similarly, C6 cells incubated with TF-Cur-Nio showed the same fluorescence distribution, suggesting that the TF can target TFR in both cell types, thereby increasing the uptake of Cur-Nio (Fig. 3E).

Our results proved that Cur-Nio and TF-Cur-Nio not only exhibited excellent biocompatibility with normal cells but also demonstrated significant cytotoxicity and pro-apoptotic effects on C6 glioma cells. Notably, TF-Cur-Nio exhibited superior performance in terms of cellular uptake, cytotoxicity, and apoptosis due to enhanced receptor-mediated endocytosis. These findings provide a solid basis for further in vivo studies and highlight the potential of TF-Cur-Nio as an effective anticancer drug delivery system.

### Study of the BBB permeability of Cur-Nio/TF-Cur-Nio

We then investigated the permeability of Cur-Nio and TF-Cur-Nio across the BBB, as the ability of drug carriers to cross the BBB is essential for effective brain tumor therapy. To begin, a BBB cell model was established using bEnd.3 cells. A schematic diagram of the bEnd.3 cell model verification experiment is shown in Fig. 3F. The concentration of sodium fluorescein in the lower chamber of the Transwell system was measured at various time points, based on a sodium fluorescein standard curve. The cumulative permeation concentration of sodium fluorescein over time was plotted, as shown in Fig. 3G. The results demonstrated that, compared to the blank control, the bEnd.3 cell model significantly limited the permeability of sodium fluorescein. The transmembrane permeability coefficient (Papp) of sodium fluorescein in the bEnd.3 cell model was calculated to be 4.3702 × 10^−6^ cm/s. This low permeability coefficient indicates that the in vitro bEnd.3 BBB model was intact and fulfilled the basic requirements of a functional blood–brain barrier.

To assess the ability of the vesicles to penetrate the BBB and reach the tumor interior, a bEnd.3 Transwell cell model was used to simulate the BBB within a tumor. Fluorescence detection was performed on the lower chamber, with the results presented in Fig. 3H. The fluorescence signal intensity of the TF-Cou6-Nio group was significantly higher than that of the Cou6-Nio group, indicating that TF could bind to the TFR on bEnd.3 cells and facilitate the passage of TF-Cur-Nio across the BBB model into the lower chamber. This confirmed that TF-Cur-Nio can traverse the BBB.

In conclusion, the TF-modified formulation demonstrated superior performance in crossing the BBB, as evidenced by its enhanced permeability in the bEnd.3 cell model. The successful permeation of TF-Cur-Nio across the BBB highlights its potential in targeted cancer therapy, particularly in overcoming the challenges associated with BBB penetration for brain tumor treatment.

### Establishment and evaluation of an in-situ C6 glioma nude mouse model

**Fig. 4:**
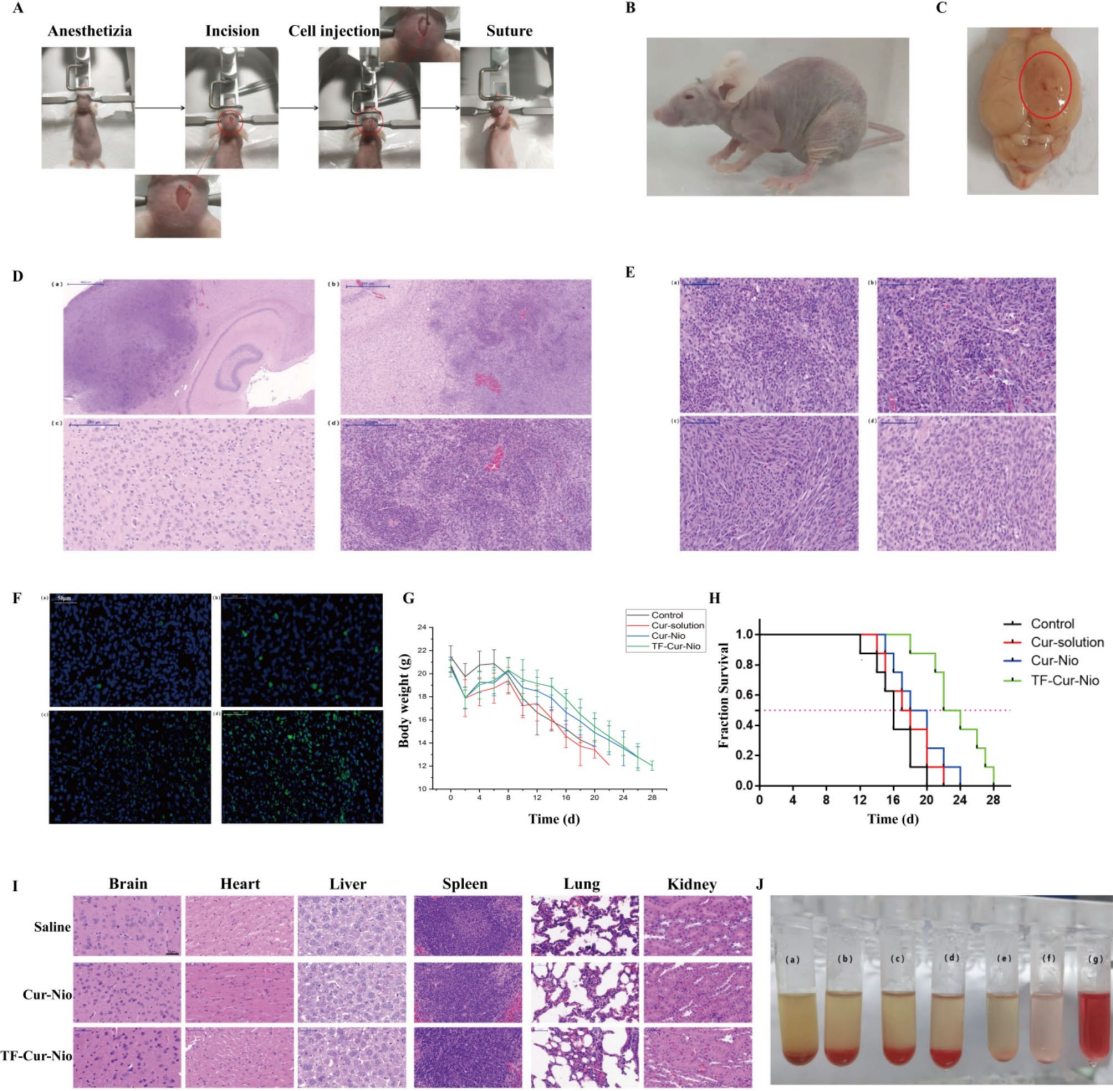
In vivo evaluation of Cur-Nio and TF-Cur-Nio. (**A**) Schematic illustration of the in-situ tumor-bearing nude mouse model. (**B**) Morphological observation of tumors in nude mice. (**C**) Gross appearance of brains from nude mice with in-situ tumors. (**D**) H&E staining of glioma tissue: (a) whole brain tissue; (b) tumor tissue with no clear boundary to normal brain tissue; (c) normal brain tissue; (d) glioma tissue. (**E**) H&E staining of glioma tissues treated with different formulations: (a) Control; (b) Cur-solution; (c) Cur-Nio; (d) TF-Cur-Nio. (**F**) TUNEL staining analysis of glioma tissues treated with different formulations: (a) Control; (b) Cur-solution; (c) Cur-Nio; (d) TF-Cur-Nio (green: apoptotic cells; blue: nucleus). (**G**) Body weight changes in C6 tumor-bearing nude mice across different treatment groups (n = 8). (**H**) Survival curves of C6 tumor-bearing nude mice in different treatment groups (n = 8). (**I**) H&E-stained sections of various tissues and organs from treated mice. (**J**) Hemolysis assay of TF-Cur-Nio solutions at different concentrations mixed with red blood cells for 4 h: (a) 200 μg/mL; (b) 100 μg/mL; (c) 50 μg/mL; (d) 25 μg/mL; (e) 10 μg/mL; (f) negative control; (g) positive control.

Having established the BBB permeability of Cur-Nio and TF-Cur-Nio, we further evaluated the anti-tumor efficacy of TF-Cur-Nio in vivo. An in-situ C6 glioma nude mouse model was established following the procedure outlined in Fig. 4A.

#### Model evaluation

The biological evaluation confirmed the model’s validity and reliability. Post-surgery, nude mice experienced a transient decrease in body weight but maintained normal diet and activity, quickly returning to baseline. One week after surgery, body weight gradually decreased, and activity levels decreased. Within two weeks, the tumor-bearing mice exhibited lethargy, delayed responses, hemiplegia, respiratory distress, and emaciation, with body weights dropping to 14 g and death occurring within 2 days (Fig. 4B).

#### Histological confirmation

On the 7th day post-implantation, the brain tissues were dissected for histological evaluation. Visual inspection revealed that the glioma tissue had grown to the surface with necrotic areas and midline displacement (Fig. 4C). Hematoxylin–eosin (H&E) staining revealed extensive proliferation of glioma cells breaching the brain surface, invasive growth without clear boundaries, and characteristic large, deeply stained nuclei, confirming the successful establishment of the in-situ C6 glioma model (Fig. 4D).

In summary, an in-situ C6 glioma nude mouse model was successfully established and validated through biological and histological assessments. This model provides a robust platform for evaluating the therapeutic potential of Cur-Nio and TF-Cur-Nio in vivo, supporting further studies in glioma therapy.

### Pharmacodynamic evaluation of Cur-Nio/TF-Cur-Nio in vivo

Based on the successful establishment of the in-situ C6 glioma nude mouse model, we proceeded to evaluate the pharmacodynamic effects of Cur-Nio and TF-Cur-Nio in vivo. This evaluation aimed to assess the therapeutic efficacy of these formulations against glioma progression.

#### Microscopic pathological changes

On the 14th day after tumor implantation, brain tissues from randomly selected tumor-bearing nude mice were dissected and subjected to H&E staining. Microscopic examination of the brain tissue pathology, as shown in Fig. 4E, revealed that in the control group, the glioma continued to deteriorate with high atypia and prominent nuclear mitotic figures. The Cur-solution group showed similar results, with increased nuclear-cytoplasmic ratios and high atypia. In the Cur-Nio group, there were fewer glioma cells with lower density and mostly oval or nearly oval nuclei. The TF-Cur-Nio group presented an increased nuclear-to-cytoplasmic ratio, with mostly oval or nearly oval nuclei, fewer glioma cells, more abundant cytoplasm, and greater differentiation. The control and Cur-solution groups presented high heterogeneity and visible mitotic figures, indicating high malignancy, whereas the TF-Cur-Nio group presented fewer tumor cells, lower cell density, and no visible mitotic figures, suggesting a better anti-glioma effect.

#### Apoptosis analysis

Apoptosis in brain tissue was evaluated via TUNEL staining, and the results were shown in Fig. 4F. The control group showed almost no apoptosis in the glioma, whereas the Cur-solution group exhibited very minimal apoptosis. The Cur-Nio group demonstrated passive targeting to the brain but insufficient penetration to induce significant glioma cell apoptosis. In contrast, the TF-Cur-Nio group exhibited a marked increase in the number of apoptotic glioma cells, highlighting its strong ability to induce apoptosis.

#### Body weight and survival analysis

After C6 glioma cell inoculation, all mice experienced a slight decrease of approximately 2 g post-inoculation, followed by recovery to their original body weight within 4 to 5 days (Fig. 4G). The first deaths occurred on day 12 in the Control group, day 14 in the Cur-solution group, day 16 in the Cur-Nio group, and day 18 in the TF-Cur-Nio group, indicating the superior anti-glioma effect of TF-Cur-Nio. Survival curves (Fig. 4H) further confirmed this trend, with median survival times of 16 days (control), 17 days (Cur-solution), 20 days (Cur-Nio), and 22 days (TF-Cur-Nio) (Table 3). Compared with Cur-solution and Cur-Nio, TF-Cur-Nio extended the median survival time by 5 and 4 days, respectively.

**Table 3:** The average survival time, median survival time and significant difference between C6 tumor-bearing nude mice in different groups (*p < 0.05, **p < 0.001, n = 9).

Group	Average survival time (d)	Median survival time (d)	Significant difference			
			Control	Cur-solution	Cur-Nio	TF-Cur-Nio
Control	16.12 ± 2.532	16				
Cur-solution	17.63 ± 2.875	17	*			
Cur-Nio	19 ± 3.071	20	**	*		
TF-Cur-Nio	23.5 ± 3.381	22	**	**	**	**

In summary, the pharmacodynamic evaluation of Cur-Nio and TF-Cur-Nio in an in-situ C6 glioma nude mouse model demonstrated that TF-Cur-Nio significantly outperformed both Cur-solution and Cur-Nio in terms of inhibiting glioma progression, inducing apoptosis, and extending survival. The enhanced therapeutic efficacy of TF-Cur-Nio can be attributed to its superior cellular uptake and targeted delivery across the BBB. These findings underscore the potential of TF-Cur-Nio as an effective treatment strategy for gliomas, providing a strong rationale for further preclinical and clinical investigations.

### Safety evaluation of Cur-Nio/TF-Cur-Nio in vivo

#### Histopathological examination

To evaluate the safety of Cur-Nio and TF-Cur-Nio, H&E-stained sections of brain, heart, liver, spleen, lung, and kidney tissues from the treated groups were examined microscopically (Fig. 4I). Compared with those in the saline control group, no significant signs of damage, necrosis, inflammation, or regenerative changes were observed in any of the tissues after tail vein injection. Specifically: (1) Brain: Tissue shows clear contours and tightly arranged cells with distinct nuclei. (2) Heart: muscle fibers were uniformly stained with no significant lesions. (3) Liver, spleen, and lung: no notable pathological changes were observed. (4) Kidney: the vesicle-treated groups exhibited smooth renal capsules, clear demarcation between the cortex and medulla, and normal, evenly distributed glomeruli in the renal cortex.

These findings indicated that neither Cur-Nio nor TF-Cur-Nio induced significant organ toxicity.

#### Hemolysis test

To further evaluate the biocompatibility of the TF-Cur-Nio formulation, a hemolysis test was conducted (Fig. 4J). After incubation at 37 °C for 4 h, the TF-Cur-Nio solution groups (a–e) maintained a consistent red blood cell (RBC) sedimentation state like that of negative control group (f), with no signs of hemolysis. In contrast, the positive control group (g) presented a clear red color, indicating significant hemolysis. The hemolysis rates for all concentrations of the TF-Cur-Nio solution were less than 5%, as presented in Table 4. This low hemolysis rate confirmed that the nanocarrier system had excellent blood compatibility and favourable biocompatibility, making it suitable for injectable administration.

**Table 4:** Hemolysis rate of TF-Cur-Nio solution with different concentrations (n = 3).

Cur concentration (μg/mL)	200	100	50	25	10
Hemolysis rate	3.69 ± 2.19	4.22 ± 0.55	0.47 ± 0.16	0.22 ± 0.22	0.23 ± 0.15

These results, combined with the superior therapeutic efficacy demonstrated in the previous sections, highlight the potential of TF-Cur-Nio as a safe and effective treatment option for gliomas, paving the way for its further development in clinical applications.

## Discussion

The treatment of intracranial diseases remains a major challenge in modern healthcare, primarily due to the restrictive nature of the BBB. Approximately 98% of small-molecule drugs and nearly all macromolecules—such as proteins, peptides, and genes—are unable to cross this barrier, significantly limiting the effectiveness of pharmacotherapy for brain disorders. Among the emerging strategies to overcome this obstacle, receptor-mediated endocytosis has shown considerable promise. Brain capillary endothelial cells express certain receptors—such as the TFR, neuropilin-1, low-density lipoprotein receptor-related protein, and N-acetylcholine receptor—on the BBB and glioblastoma multiforme cells, while these receptors are either minimally expressed or absent in normal tissues. Therefore, engineering nanocarriers with ligands that specifically target these receptors can substantially enhance drug delivery across the BBB, improve anticancer efficacy, and reduce off-target effects.

This study introduced an innovative approach involving the use of Span 60 and cholesterol to formulate Cur-Nio, enhancing the metabolic stability and bioavailability of Cur and significantly extending its circulation time in vivo. Through a physical method, we successfully incorporated TF onto the vesicle surface, enabling targeted delivery to the brain. Additionally, we developed comprehensive in vitro and in vivo models to assess the targeted delivery, therapeutic efficacy, and biocompatibility of the TF-Cur-Nio system. The results from both in vitro and in vivo studies confirmed the system’s ability to achieve targeted delivery, demonstrate therapeutic efficacy, and exhibit biocompatibility, thereby offering a promising new strategy for the targeted treatment of gliomas.

Despite these encouraging findings, several areas warrant further investigation. For instance, incorporating charge-inducing agents could improve physical stability and optimize the charge characteristics of the formulation, while PEGylation might impart stealth properties that further extend circulation time. Future research should also expand beyond the current pharmacodynamic assessments to include comprehensive pharmacokinetic studies, which would provide crucial insights into the systemic distribution and bioavailability of the drug. Moreover, a more detailed exploration of the antitumor mechanisms of Cur in glioma cells is needed. Considering the heterogeneity of receptor expression in gliomas, developing vesicle systems with dual-targeting capabilities may engage multiple pathways simultaneously, potentially yielding superior therapeutic outcomes.

In summary, while nonionic surfactant vesicles offer significant advantages over traditional liposomes, their application in brain drug delivery remains relatively underexplored. Although our nanovesicles have demonstrated promising efficacy in both cellular models and tumor-bearing nude mice, their potential toxicity, stability, and the mechanisms underlying drug-vesicle interactions and release require further elucidation. Addressing these challenges through in-depth research will be crucial for optimizing the physicochemical properties of these vesicles and ensuring effective and safe in vivo drug delivery.

## Methods

### Cell culture

The rat glioma cell line C6 was obtained from Wuhan Procell Life Science & Technology Co., Ltd., and maintained in DMEM supplemented with 10% FBS and 1% penicillin-streptomycin. The mouse brain microvascular endothelial cell line bEnd.3 was acquired from Beina Biological Cell Bank, and cultured in DMEM containing 10% FBS and 1% penicillin-streptomycin. All the cells were cultured at 5% CO2, 37 °C.

### Animals

BALB/c immunodeficient nude mice, male, weighing 20-25 g, were purchased from Beijing Vital River Laboratory Animal Technology Co., Ltd. Male SD rats, weighing 200-230 g, were purchased from Beijing Vital River Laboratory Animal Technology Co., Ltd. Male KM mice, weighing 20-25 g, were purchased from Beijing Vital River Laboratory Animal Technology Co., Ltd.

All animals were housed at the Institute of Toxicology and Pharmacology, Academy of Military Medical Sciences. All in vivo experimental protocols were approved by the Institutional Animal Care and Use Committee of the Institute of Pharmacology and Toxicology, Beijing.

### High performance liquid chromatography (HPLC)

Quantification of Cur was performed using an Agilent 1200 Series HPLC system (Agilent Technologies, Santa Clara, CA, USA). The chromatographic separation was achieved on an E-clipse XDB-C18 column (250 mm × 4.6 mm, 5 μm; Agilent Technologies) maintained at 30 °C. The mobile phase consisted of acetonitrile and 4% glacial acetic acid in a ratio of 55:45, running at a flow rate of 1 mL/min. Detection was carried out at a wavelength of 428 nm, with an injection volume of 10 μL.

### Preparation of Cur-Nio/TF-Cur-Nio

Cur-Nios were prepared using the thin-film dispersion-ultrasonication method. Appropriate amounts of Span 60, cholesterol, and curcumin (Cur) raw material were dissolved in an organic solvent mixture (chloroform: anhydrous ethanol = 4:1) at the bottom of a round-bottom flask. The solution was rotary evaporated under reduced pressure until a thin film formed at the bottom of the flask, followed by overnight drying in a vacuum drying oven. Ultrapure water was then added to hydrate the film. After hydration, the mixture was probe-sonicated and filtered through 0.45 μm and 0.22 μm filters to obtain the Cur-Nios. The preparation process for blank vesicles was identical to that of Cur-Nios, except that curcumin was not added.

To prepare TF-Cur-Nio, the Cur-Nio suspension was transferred to a glass vial, followed by the addition of a 1 mg/mL TF standard solution to achieve a final TF concentration of 50 μg/mL. The mixture was then subjected to ultrasonication using a probe sonicator set at 20% amplitude, operating in 2-second on/off cycles for a total of 6 min (three cycles of 2 min each, with a 2-min rest between cycles). The resulting TF-Cur-Nio suspension was enclosed in a dialysis bag, sealed, and immersed in 500 mL of distilled water. Dialysis was carried out under continuous stirring at a controlled temperature for 24 h to remove unbound TF, yielding the final TF-Cur-Nio formulation.

TF conjugation efficiency was quantified using a BCA assay. A TF standard curve (2.5-100 μg/mL) was established. Unbound TF was collected from the dialysis filtrate and analyzed via the same method, and the conjugation efficiency was calculated as follows:

1 $${\text{Conjugation}}\,{\text{Efficiency}}\,(\% ) = \left( {{\text{Total}}\,{\text{TF}} - {\text{Free}}\,{\text{TF}}} \right)/{\text{Total}}\,{\text{TF}} \times 100\%$$

The particle size, PDI, and zeta potential of TF-Cur-Nios were then measured using a Nano-ZS90 particle size analyzer. The appearance of TF-Cur-Nios was observed through TEM.

### Single-factor experiments

To conduct single-factor experiments while keeping all other formulation and process conditions constant, the encapsulation efficiency was used as the evaluation index to investigate the impact of different mass ratios of Span 60 to cholesterol (1:1, 2:1, 3:1, 4:1, 5:1), different mass ratios of cholesterol to curcumin (2:1, 4:1, 6:1, 8:1, 10:1), varying volumes of hydration liquid (5, 10, 15, 20, 30 mL), different hydration times (0.5, 1, 1.5, 2, 2.5 h), different hydration temperatures (30, 40, 50, 60, 70 °C), and varying ultrasonication times (2, 4, 6, 8, 10 min) on the encapsulation efficiency of Cur-Nios, aiming to optimize the preparation process and formulation.

Subsequently, a CCD-RSM was employed. Based on the single-factor results, the mass ratio of Span 60 to cholesterol, hydration time, and hydration volume were selected as key factors. With encapsulation efficiency, drug-loading capacity, and average particle size as evaluation criteria, a three-factor five-level CCD-RSM experiment was designed. Data was analyzed using statistical software to optimize and determine the final preparation conditions.

### Encapsulation assessment

The EE of Cur-Nio was determined using low-speed centrifugation and filtration. A 2 mL sample of the Cur-Nio suspension was centrifuged at 3000 rpm for 10 min. Due to its small size, Cur-Nio remains suspended, while Cur, which is insoluble in water, precipitates. The supernatant was filtered through 0.45 μm and 0.22 μm filters to remove unencapsulated Cur. Then, 1 mL of the filtrate was taken, diluted with anhydrous ethanol, vortexed, and sonicated for 30 min to break the emulsion. The Cur content was measured by HPLC. The EE and DL were calculated as follows:

2 $${\text{EE}}\,(\% ) = {\text{m}}1/{\text{m}}2 \times 100\%$$

3 $${\text{DL}}\,(\% ) = {\text{m}}1/{\text{mtotal}} \times 100\%$$

### In vitro release study

A dynamic dialysis method was performed to evaluate the in vitro release of TF-Cur-Nios. A release medium consisting of 0.5% SDS in a phosphate buffer at pH 5 was used to simulate the release behavior of TF-Cur-Nios within tumor cell lysosomes. An appropriate amount of TF-Cur-Nio suspension and an equivalent concentration of curcumin solution prepared in propylene glycol were separately placed into dialysis bags with a molecular weight cut-off (MWCO) of 8000 Da. The ends of the dialysis bags were sealed and then immersed in centrifuge tubes containing 30 mL of the release medium. The release study was conducted at 37 °C with a shaking speed of 100 rpm. Samples of 1.5 mL were taken at predetermined intervals (0.5, 1, 2, 4, 8, 12, 24, 48, and 72 h), and the same volume of fresh, pre-warmed release medium was replenished immediately. The samples were filtered through a 0.22 μm microporous membrane and analyzed by HPLC. The cumulative release was calculated as follows:

4 $${\text{Cumulative}}\,{\text{Release}}\,{\text{(\% )}} = \left( {\sum {\text{Released}}\,{\text{Amount}}\,{\text{at}}\,{\text{time}}\,{\text{ti}}/{\text{Initial}}\,{\text{Loaded}}\,{\text{Amount}}} \right) \times 100\%$$

∑Released Amount at time ti is the cumulative amount of drug released at each time point ti. Initial Loaded Amount is the total amount of drug initially loaded into the delivery system.

### Cytotoxicity assay

Cur was dissolved in DMSO and diluted in DMEM to ensure the final DMSO concentration did not exceed 0.5%. Cur, Cur-Nio, and TF-Cur-Nio were prepared in DMEM at concentrations of 30, 20, 10, 5, 2.5, and 1 μg/mL, with six replicate wells for each concentration. C6 glioma and bEnd.3 cells were co-incubated with these samples for 24 h. After incubation, the medium was replaced with DMEM containing 5 mg/mL MTT (1 mL per well), and plates were incubated for 4 h. The medium was then removed, and 100 μL of DMSO was added to each well to dissolve the formazan crystals, followed by shaking for 10 min. Absorbance was measured at 490 nm using a microplate reader to determine the half-maximal inhibitory concentration (IC_50_) for each group.

### Apoptosis study

Based on the MTT assay results, solutions of Cur, Cur-Nio, and TF-Cur-Nio were diluted in DMEM to their respective lowest IC_50_ values. Negative control groups were also established. Each treatment was added to the wells of a 6-well plate and incubated with C6 cells for 24 h.

Following incubation, C6 cells were collected into centrifuge tubes and resuspended in 100 μL of 1× Binding Buffer. To each suspension, 5 μL of Annexin V-FITC was added and incubated in the dark for 10 min. Next, 5 μL of propidium iodide (PI) staining solution was added, followed by an additional 15-min incubation in the dark. Cell apoptosis was then quantified using a flow cytometer.

### Cellular uptake study

To assess the time- and concentration-dependent uptake of Cur-Nio and TF-Cur-Nio by C6 cells, a Cur fluorescence standard curve was established by preparing a series of curcumin solutions and measuring their fluorescence intensity. Logarithmically growing C6 cells were seeded at a density of 1 × 10^5^ cells per well in 12-well plates and co-incubated with vesicle solutions at different concentrations (2.5–20 μg/mL) or for varying time periods (0.5–4 h). After incubation, cells were washed with PBS and lysed with RIPA buffer containing PMSF. The lysates were collected, centrifuged, and the protein concentration in the supernatant was determined. Cur uptake was calculated via fluorescence detection and standardized to protein content.

### Fluorescence co-localization evaluation

Due to the weak fluorescence intensity and susceptibility to photobleaching of Cur, coumarin-6 (Cuo6) was used as a substitute due to its structural similarity to curcumin. Therefore, Cuo6-loaded Nios (Cuo6-Nio) and TF-modified Cuo6-Nios (TF-Cuo6-Nio) were prepared using the thin-film dispersion-ultrasonication method for fluorescence co-localization analysis of cell uptake. C6 and bEnd.3 cells were seeded at 1 × 10^5^ cells per well in confocal dishes, incubated overnight, and then treated with the Cuo6-Nio or TF-Cuo6-Nio solutions. After 1 h of dark incubation, cells were washed with PBS, fixed with 4% paraformaldehyde, stained with Hoechst 33258, and observed under a confocal microscope.

### BBB model establishment and permeability assessment

The bEnd.3 cell monolayer model was established to simulate the BBB in vitro by seeding 1 × 10^4^ logarithmically growing bEnd.3 cells per well in 24-well Transwell inserts. Medium changes and TEER measurements were performed every other day. After 7 days, the model was validated through a 4-hour leakage test, TEER value measurement, and sodium fluorescein permeability test to assess barrier function and integrity.

For permeability studies, validated models were rinsed with pre-warmed medium, and Cur-Nio and TF-Cur-Nio solutions were added. Samples from the lower chamber were collected at intervals (0, 30, 60, 90, 120, 150, 180, and 210 min), replaced with fresh DMEM, and analyzed for Cur fluorescence to evaluate vesicle permeability across the BBB.

### In-situ C6 glioma mouse model establishment

To establish an in-situ C6 glioma mouse model, harvest C6 cells, resuspend them in 50 μL PBS, and inject 5 μL (1 × 10^6^ cells) into the brains of anesthetized BALB/c nude mice using a stereotactic apparatus. Post-surgery, monitor recovery and maintain the mice on a warming blanket. Evaluate the model by weighing the mice every other day, inspecting brain tumors for size and location on day 7, and preparing 5 μm paraffin sections of the brain tissue for (H&E staining and microscopic examination to assess the pathological condition.

### Pharmacodynamics study of Cur-Nio/TF-Cur-Nio

Establish in-situ C6 glioma in nude mice and divide them into four groups (n = 9): saline control, Cur-solution, Cur-Nio, and TF-Cur-Nio. Administer treatments via tail vein injection on day 2, 4, 6, 8, 10, and 12 post-tumor implantations, with a Cur dose of 2 mg/kg, totaling six administrations. Evaluate the therapeutic efficacy of the vesicles through the following methods: (1) H&E Staining: One day after the final treatment, randomly select mice from each group, extract brain tissues, fix them in 4% paraformaldehyde for 24 h, dehydrate, embed in paraffin, and prepare 5 μm sections. Perform H&E staining and examine the pathological condition under a microscope. (2) TUNEL Assay: Assess apoptosis in C6 glioma cells using TUNEL staining. 1 day after the last treatment, select one mouse per group, extract brain tissues, fix them in 4% paraformaldehyde, prepare paraffin sections, deparaffinize, rehydrate, increase permeability with proteinase K, incubate with TUNEL reaction mixture, counterstain nuclei with DAPI, mount slides, and observe under a fluorescence microscope. (3) Weight and Survival Analysis: Regularly measure and plot the body weight changes of tumor-bearing mice. Record survival times until natural death and plot survival curves to calculate the mean survival time and median survival for each group.

To evaluate the safety of Cur-Nio and TF-Cur-Nio, KM mice were divided into three groups (n = 9): saline control, Cur-Nio, and TF-Cur-Nio. Treatments were administered via tail vein injection every other day at 2 mg/kg for six doses. One day post-final treatment, brain, heart, liver, spleen, lung, and kidney tissues were collected, fixed in 4% paraformaldehyde, processed into paraffin sections, stained with H&E, and examined microscopically for histopathological changes.

### Safety evaluation of Cur-Nio/TF-Cur-Nio

For hemolytic activity assessment, fresh arterial blood from SD rats was used to prepare a 2% RBC suspension (n = 3). TF-Cur-Nio was diluted to Cur concentrations of 200, 100, 50, 25, and 10 μg/mL, mixed with RBC suspensions, and incubated at 37 °C for 4 h. After centrifugation, supernatants were analyzed for hemolysis at 545 nm, using sterile water as the positive control and saline as the negative control (n = 3). The hemolysis rate was calculated as:

5 $${\text{Hemolysis}}\,{\text{Rate}}\,(\% ) = ({\text{A}}_{{{\text{sample}}}} - {\text{A}}_{{{\text{blank}}}} )/({\text{A}}_{{{\text{positive}}}} - {\text{A}}_{{{\text{blank}}}} ) \times 100\%$$

This integrated safety evaluation assessed both organ toxicity and hemolytic potential, providing critical data on the biocompatibility and safety of Cur-Nio and TF-Cur-Nio formulations.

### Statistical analysis method

Data was analyzed using GraphPad Prism 7 software. Results are expressed as mean ± SD. Differences between groups were compared using t-tests. A *p* value < 0.05 indicates statistical significance, and *p *< 0.01 indicates highly significant differences.

## Data Availability

The datasets used and/or analysed during the current study available from the corresponding author on reasonable request.
